# Proxalutamide reduces SARS-CoV-2 infection and associated inflammatory response

**DOI:** 10.1073/pnas.2221809120

**Published:** 2023-07-17

**Authors:** Yuanyuan Qiao, Jesse W. Wotring, Yang Zheng, Charles J. Zhang, Yuping Zhang, Xia Jiang, Carla D. Pretto, Sanjana Eyunni, Abhijit Parolia, Tongchen He, Caleb Cheng, Xuhong Cao, Rui Wang, Fengyun Su, Stephanie J. Ellison, Yini Wang, Jun Qin, Honghua Yan, Qianxiang Zhou, Liandong Ma, Jonathan Z. Sexton, Arul M. Chinnaiyan

**Affiliations:** ^a^Michigan Center for Translational Pathology, University of Michigan, Ann Arbor, MI 48109; ^b^Department of Pathology, University of Michigan, Ann Arbor, MI 48109; ^c^Rogel Cancer Center, University of Michigan, Ann Arbor, MI 48109; ^d^Department of Medicinal Chemistry, College of Pharmacy, University of Michigan, Ann Arbor, MI 48109; ^e^Department of Internal Medicine, University of Michigan, Ann Arbor, MI 48109; ^f^State Key Laboratory of Proteomics, Beijing Proteome Research Center, National Center for Protein Sciences (Beijing), Beijing Institute of Lifeomics, Beijing 102206, China; ^g^Kintor Pharmaceutical Limited, Suzhou Industrial Park, Suzhuo 215123, China; ^h^Center for Drug Repurposing, University of Michigan, Ann Arbor, MI 48109; ^i^Michigan Institute for Clinical and Health Research, University of Michigan, Ann Arbor, MI 48109; ^j^Department of Pharmacology, University of Michigan, Ann Arbor, MI 48109; ^k^HHMI, University of Michigan, Ann Arbor, MI 48109; ^l^Department of Urology, University of Michigan, Ann Arbor, MI 48109

**Keywords:** proxalutamide, SARS-CoV-2, COVID-19, androgen receptor, cytokines

## Abstract

Early in the COVID-19 pandemic, data suggested that males had a higher risk of developing severe disease and that androgen deprivation therapy might be associated with protection. Combined with the fact that *TMPRSS2* (*transmembrane serine protease 2*), a host entry factor for the SARS-CoV-2 virus, was a well-known androgen-regulated gene, this led to an upsurge of research investigating androgen receptor (AR)-targeting drugs. Proxalutamide, an AR antagonist, was shown in initial clinical studies to benefit COVID-19 patients; however, further validation is needed as one study was retracted. Due to continued interest in proxalutamide, which is in phase 3 trials, we examined its ability to impact SARS-CoV-2 infection and downstream inflammatory responses. Proxalutamide exerted similar effects as enzalutamide, an AR antagonist prescribed for advanced prostate cancer, in decreasing AR signaling and expression of *TMPRSS2* and *angiotensin-converting enzyme 2 (ACE2)*, the SARS-CoV-2 receptor. However, proxalutamide led to degradation of AR protein, which was not observed with enzalutamide. Proxalutamide inhibited SARS-CoV-2 infection with an IC_50_ value of 97 nM, compared to 281 nM for enzalutamide. Importantly, proxalutamide inhibited infection by multiple SARS-CoV-2 variants and synergized with remdesivir. Proxalutamide protected against cell death in response to tumor necrosis factor alpha and interferon gamma, and overall survival of mice was increased with proxalutamide treatment prior to cytokine exposure. Mechanistically, we found that proxalutamide increased levels of NRF2, an essential transcription factor that mediates antioxidant responses, and decreased lung inflammation. These data provide compelling evidence that proxalutamide can prevent SARS-CoV-2 infection and cytokine-induced lung damage, suggesting that promising clinical data may emerge from ongoing phase 3 trials.

## Significance

Drugs that target androgen receptor (AR) signaling, including those that inhibit production of androgen ligands (degarelix) and those that bind to and directly block AR activity (enzalutamide), have been investigated in clinical trials for the treatment of COVID-19 but failed to produce positive results. Another AR antagonist, proxalutamide, is in ongoing phase 3 studies for COVID-19 after showing initial positive findings. Data from this study show that proxalutamide can inhibit infection of multiple variants of SARS-CoV-2 in vitro. These data suggest that proxalutamide should continue to be investigated in clinical studies as a potential therapy for COVID-19.

Over 3 y have passed since the first documented cases of COVID-19 arose from infection by the severe acute respiratory syndrome coronavirus 2 (SARS-CoV-2), yet many challenges remain worldwide in preventing and treating the disease (1). Robust vaccination campaigns led to rapid development, testing, and deployment of several vaccines effective against infection and serious illness from the initial SARS-CoV-2 genetic lineages (2–6). However, as the pandemic continued, waning vaccine protection and emergence of new variants have led to breakthrough infections, as well as many people now having been infected multiple times (5–9). Booster vaccines, including bivalent boosters effective against the highly transmissible omicron variant, have been developed in an effort to overcome these challenges (10). Oral antivirals such as molnupiravir and nirmatrelvir–ritonavir have been developed for high-risk individuals who contract COVID-19, but these are also met with obstacles like potential recurrent infections or contraindications with other commonly prescribed drugs (11–14). Together, these challenges highlight the ongoing critical need for new therapeutics to combat SARS-CoV-2.

As it is the initial step in the viral life cycle, the entry process has been intensely studied to understand how to potentially block SARS-CoV-2 infection (15). Early data during the pandemic showed that the spike (S) protein of SARS-CoV-2 binds to host angiotensin-converting enzyme 2 (ACE2) receptors on the cell surface to initiate entry (16, 17). Cleavage of the spike protein by transmembrane serine protease 2 (TMPRSS2) facilitates fusion of the viral and cell membranes and cell entry (18, 19). With the presumed advantage that it will be difficult for the virus to mutate and evade host-directed drugs, multiple preclinical and clinical research efforts have since followed examining the efficacy of therapies directly targeting TMPRSS2 and ACE2, albeit with mixed results and several studies still ongoing (20–25).

Since *TMPRSS2* is a well-known androgen receptor (AR)-regulated gene, early hypotheses suggested that inhibition of AR activity could be a potential treatment strategy for COVID-19 (26). As demographic data became available, many reports also observed that males had higher incidences of severe SARS-CoV-2 infections that required intensive care unit (ICU) admission or resulted in death (27–29). In further support of the initial hypothesis that AR activity may drive COVID-19 pathogenesis, a retrospective study during the first months of the pandemic observed a reduced incidence of SARS-CoV-2 infections in prostate cancer patients taking androgen deprivation therapy (ADT) compared to those not receiving ADT (30). Other small studies supported this observation and the premise that anti-androgens could be protective against severe COVID-19 (31, 32); however, these results quickly became debated as other studies found no association between ADT and SARS-CoV-2 infectivity (33–35).

These preliminary observations prompted a burst of basic science and clinical studies to attempt to elucidate the role of androgens in SARS-CoV-2 infection and determine whether AR inhibitors could be viable treatment options for COVID-19. Studies with AR antagonists prescribed for prostate cancer treatment (e.g., enzalutamide, apalutamide, and darolutamide) have since shown that SARS-CoV-2 infectivity can be decreased in vitro in certain contexts with these drugs (36–38). However, some randomized, controlled clinical trials of AR inhibition in COVID-19 patients have not produced encouraging results. For instance, in the Hormonal Intervention for the Treatment in Veterans with COVID-19 Requiring Hospitalization (HITCH) trial (NCT04397718) which tested degarelix, a gonadotropin-releasing hormone (GnRH) antagonist that rapidly suppresses testosterone levels, in male veterans hospitalized with COVID-19, no improvement in clinical outcome was observed compared to placebo (39). Similarly, the COVIDENZA trial (NCT04475601) found no improvement in outcome of COVID19-positive male or female patients who were randomized to treatment with enzalutamide vs. standard of care (40).

In contrast, the AR antagonist proxalutamide was also tested as a possible treatment for COVID-19 in randomized, controlled trials and showed encouraging positive benefits (41–43), but these findings were met with caution from the scientific community after a retraction statement was issued for one of the publications, citing concerns over randomization (44). Proxalutamide is currently in additional phase 3 trials for COVID-19 in both outpatient (NCT04870606 and NCT04869228) and hospital (NCT05009732) settings in different countries, including the United States. Proxalutamide was originally developed as an AR antagonist for advanced prostate cancer and is in ongoing phase 2 clinical trials for this indication as well (45–47). Our previous study found that AR antagonists (enzalutamide, apalutamide, and darolutamide) and degraders decreased *TMPRSS2* and *ACE2* expressions and were potent inhibitors of SARS-CoV-2 infectivity in vitro (37). Given these data and the continued clinical interest surrounding proxalutamide in COVID-19, we sought to test proxalutamide for its ability to impact SARS-CoV-2 infection. We find that proxalutamide inhibits cellular infection by multiple SARS-CoV-2 variants and shows synergistic activity in vitro with remdesivir, an antiviral demonstrated to have clinical benefit in COVID-19 patients (48, 49). Additionally, in vivo studies showed that prophylactic treatment with proxalutamide can improve overall survival in mouse models of the TNFα (tumor necrosis factor alpha) and IFNγ (interferon gamma)-induced cytokine storm triggered by SARS-CoV-2 infection (50), potentially occurring through increases in the nuclear factor erythroid 2-related factor 2 (NRF2) transcription factor responsible for mediating cellular antioxidant responses. Altogether, this study provides characterization of proxalutamide in SARS-CoV-2 infection models and provides data to possibly explain positive results that may emerge from clinical trials of proxalutamide for COVID-19 treatment.

## Results

**Fig. 1.**
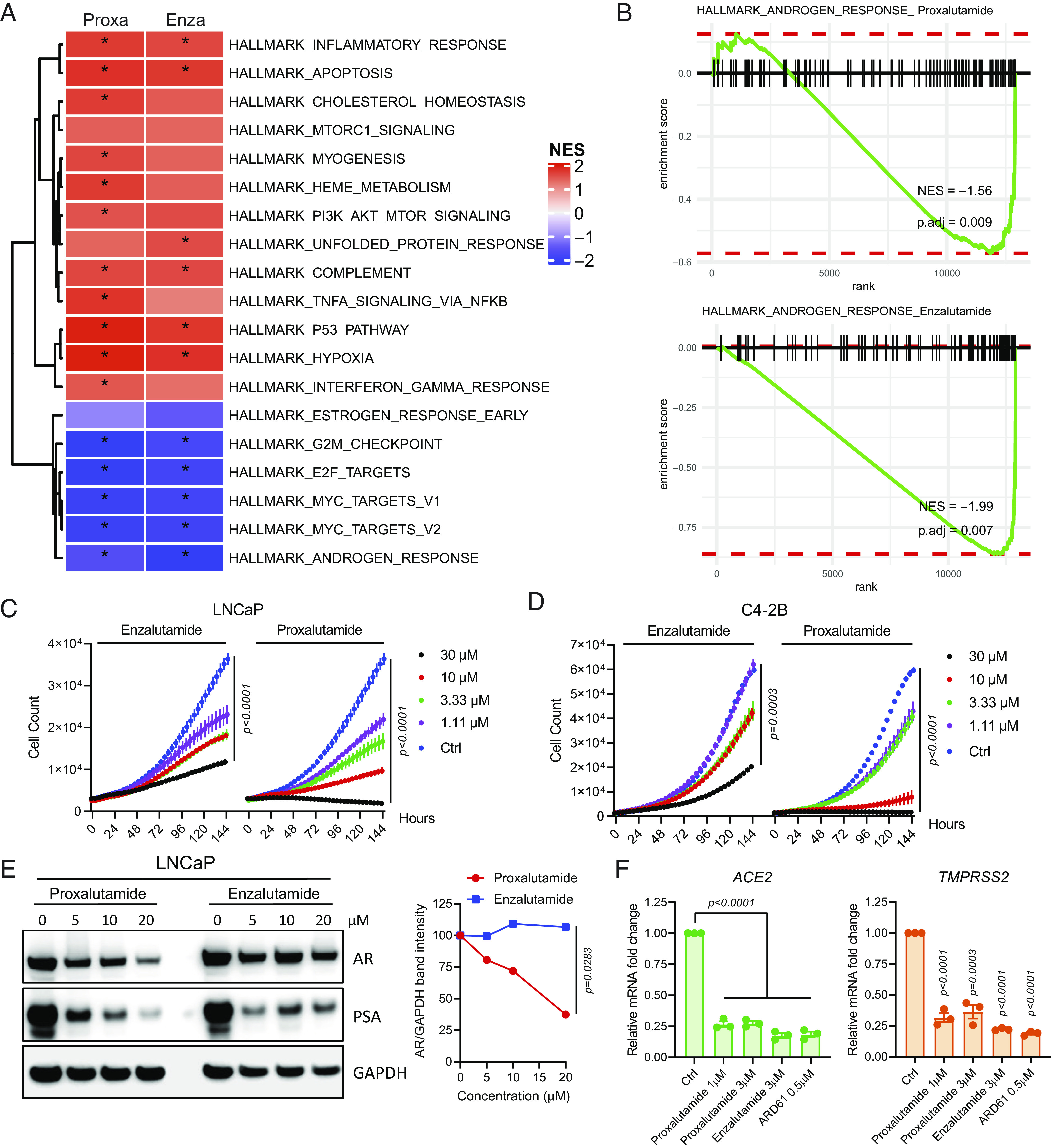
Proxalutamide is a recently developed AR antagonist that also down-regulates AR protein levels. (*A*) Hallmark of differential expressed gene signatures in proxalutamide (Proxa) and enzalutamide (Enza) treatment vs. control in LNCaP cells; the asterisk indicates a *P* value of less than 0.01. (*B*) Gene set enrichment of the androgen response pathway in proxalutamide- or enzalutamide-treated LNCaP cells. (*C*) Cell growth inhibition in enzalutamide- or proxalutamide-treated LNCaP cells. Ctrl, control. *P* values were calculated by the two-tailed unpaired *t* test between ctrl and 30 µM enzalutamide or proxalutamide (not between each dose). (*D*) Cell growth inhibition in enzalutamide- or proxalutamide-treated C4-2B cells. Ctrl, control. *P* values were calculated by the two-tailed unpaired *t* test between ctrl and 30 µM enzalutamide or proxalutamide (not between each dose). (*E*) Immunoblotting of AR and PSA protein in LNCaP cells after treatment with various concentrations of proxalutamide and enzalutamide for 24 h. Quantification of band intensity of AR/GAPDH is shown on the right. *P* values were calculated by the two-tailed unpaired *t* test between 20 µM proxalutamide and enzalutamide. (*F*) Relative mRNA expression of *ACE2* and *TMPRSS2* in LNCaP cells after the indicated treatment. *P* values were calculated by the two-tailed unpaired *t* test between control and the indicated treatment.

Proxalutamide is an AR antagonist recently developed for castration-resistant prostate cancer (CRPC) (47), in comparison to enzalutamide which has been commonly prescribed for CRPC treatment for several years (51). To first compare the transcriptomic changes associated with proxalutamide and enzalutamide, RNA-sequencing (RNA-Seq) analysis was carried out in AR-positive prostate cancer Lymph Node Carcinoma of the Prostate (LNCaP) cells using either 20 µM proxalutamide or enzalutamide for 8 h of treatment. Gene set enrichment analysis was achieved by examining differentially expressed genes in either proxalutamide- or enzalutamide-treated cells compared to control. The normalized enrichment score results indicated that androgen responses were the top down-regulated hallmark in both proxalutamide- and enzalutamide-treated LNCaP cells (Fig. 1*A*). Gene set enrichment analysis on androgen responses further confirmed that proxalutamide significantly down-regulated androgen-regulated genes that were suppressed by enzalutamide (Fig. 1*B*), suggesting proxalutamide suppresses AR signaling. In addition, the effect of proxalutamide on cell proliferation was examined in LNCaP cells and a castration-resistant variant of LNCaP called C4-2B cells. In both LNCaP and C4-2B cells, proxalutamide and enzalutamide treatment resulted in dose-dependent inhibition of cell proliferation in vitro, but growth inhibition was greater with proxalutamide treatment compared to enzalutamide at the same concentrations (Fig. 1 *C* and *D*). Importantly, we found that proxalutamide not only suppressed AR signaling but also decreased AR protein levels, which were not altered by enzalutamide treatment (Fig. 1*E*), indicating that proxalutamide possesses stronger inhibition of the AR signaling pathway than enzalutamide. Previously, we reported that enzalutamide can transcriptionally down-regulate SARS-CoV-2 entry factors *TMPRSS2* and *ACE2* (37). Here, we found that proxalutamide had the same ability to decrease *TMPRSS2* and *ACE2* (Fig. 1*F*). Thus, we postulated that proxalutamide may block SARS-CoV-2 infection.

**Fig. 2.**
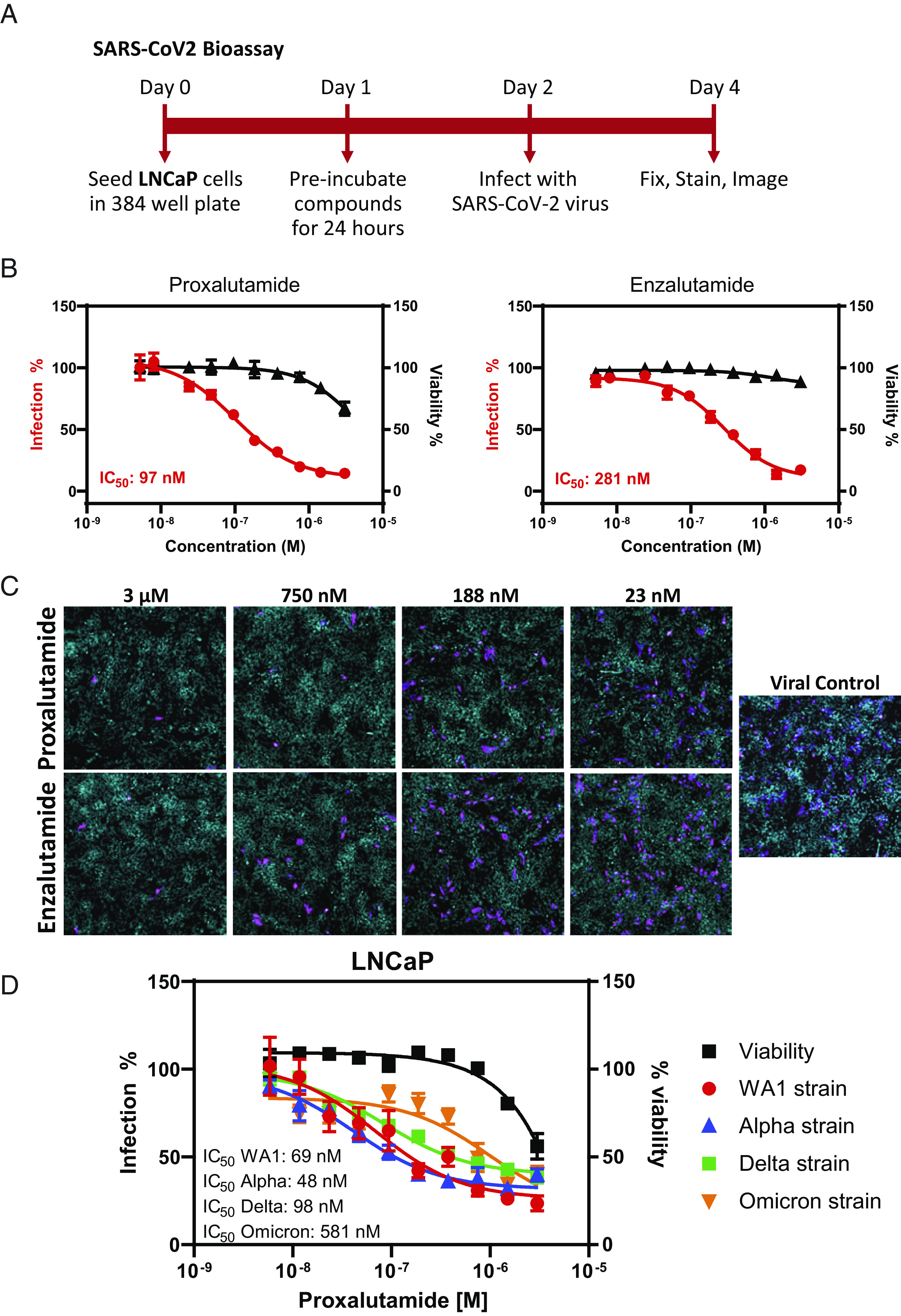
Proxalutamide inhibits multiple strains of SARS-CoV-2 infection in vitro. (*A*) Schematic illustration of the SARS-CoV-2 bioassay. (*B*) Dose-dependent inhibition of SARS-CoV-2 WA1 strain infection by proxalutamide and enzalutamide in LNCaP cells with IC_50_ values shown for each. Cell viability is also graphed. (*C*) Representative images of SARS-CoV-2 WA1 strain infection after proxalutamide or enzalutamide treatment in LNCaP cells. (*D*) Dose-dependent inhibition of infection by multiple strains of SARS-CoV-2 with proxalutamide treatment in LNCaP cells.

Employing a SARS-CoV-2 bioassay platform, we have established an in vitro system with which to examine the various strains of authentic SARS-CoV-2 viral infection (37, 52). In this system, cells were pretreated with the experimental compounds for 24 h prior to SARS-CoV-2 infection for an additional 72 h (Fig. 2*A*). The results showed that proxalutamide decreased cellular infection by the WA1 strain of SARS-CoV-2 in a dose-dependent manner with an IC_50_ value of 97 nM, whereas enzalutamide decreased infectivity with an IC_50_ value of 281 nM (Fig. 2*B*). Representative images of cellular infectivity by the WA1 strain of SARS-CoV-2 in control-, proxalutamide-, or enzalutamide-treated conditions confirmed that decreased infection could be achieved by the AR antagonists proxalutamide and enzalutamide in LNCaP cells (Fig. 2*C*). Since several variants of the SARS-CoV-2 virus have emerged throughout the pandemic, we examined the effect of proxalutamide against infection of multiple strains. The results indicated that proxalutamide possessed robust inhibitory effects in blocking SARS-CoV-2 infection by the most common strains, including WA1, alpha, delta, and omicron, with IC_50_ values of 69 nM, 48 nM, 98 nM, and 581 nM, respectively, in LNCaP cells (Fig. 2*D*).

**Fig. 3.**
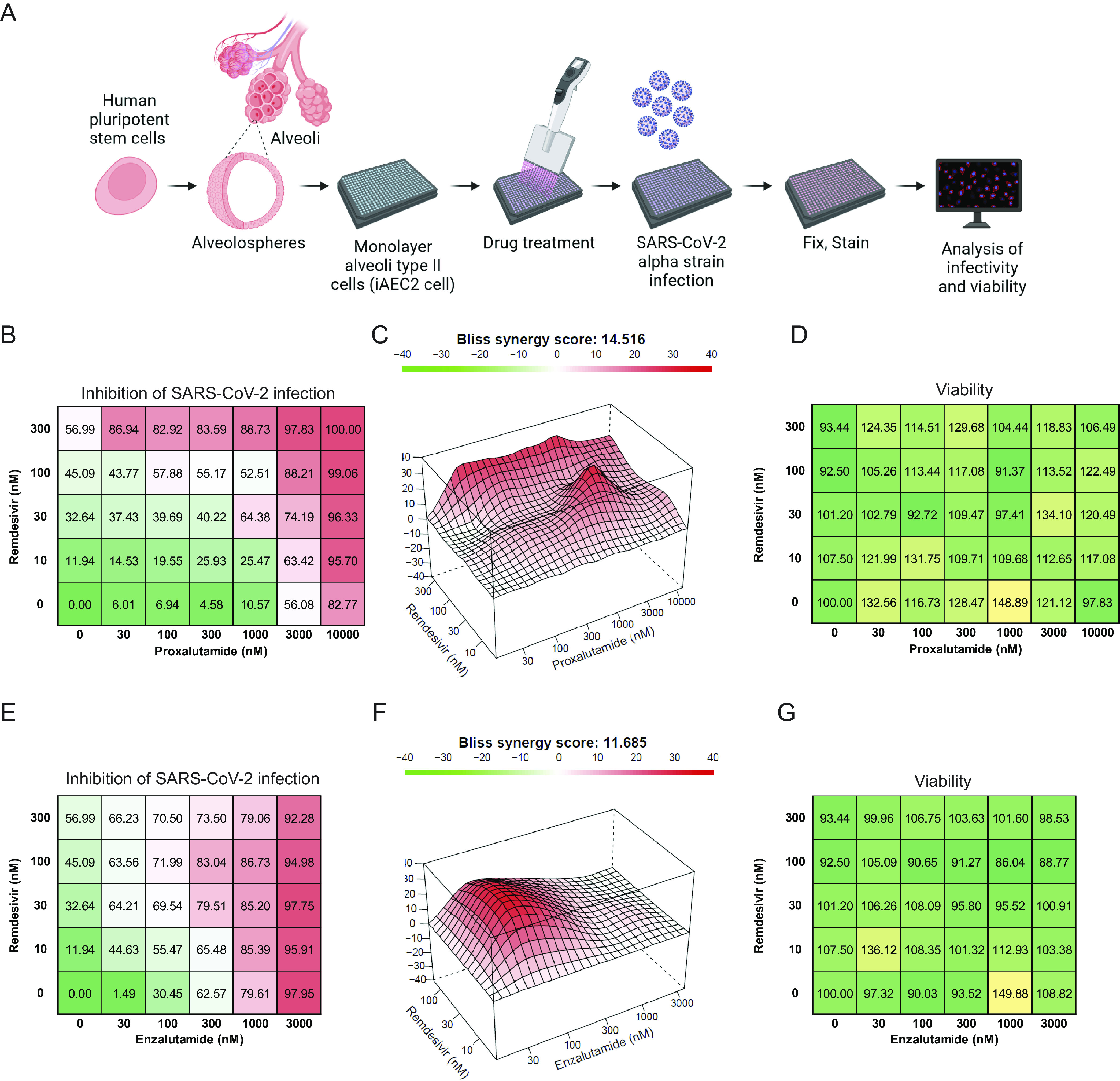
Proxalutamide and remdesivir combination exerts strong synergistic effect in blocking SARS-CoV-2 infection in iAEC2. (*A*) Schematic illustration of the study design of the SARS-CoV-2 bioassay on iAEC2 cells. (*B*) Combination matrix of proxalutamide and remdesivir in inhibition of SARS-CoV-2 alpha strain infection. (*C*) Bliss synergy score of proxalutamide and remdesivir against SARS-CoV-2 alpha strain infection. (*D*) Combination matrix of cell viability on proxalutamide and remdesivir. (*E*) Combination matrix of enzalutamide and remdesivir in inhibition of SARS-CoV-2 alpha strain infection. (*F*) Bliss synergy score of enzalutamide and remdesivir against SARS-CoV-2 alpha strain infection. (*G*) Combination matrix of cell viability on enzalutamide and remdesivir.

Furthermore, remdesivir is a Food and Drug Administration (FDA)-approved agent for treatment of SARS-CoV-2 infection (48, 49). The combinatorial effect of proxalutamide or enzalutamide and remdesivir in preventing infection by the SARS-CoV-2 alpha strain was examined in induced human alveolar cells (iAEC2) (Fig. 3*A*). The results indicated that proxalutamide had a strong synergistic effect with remdesivir in inhibition of alpha strain infection and achieved 100% protection against infection (Fig. 3*B*), with a synergy score of 14.516 (Fig. 3*C*). Similarly, the enzalutamide and remdesivir combination achieved synergy but with a slightly weaker synergistic effect than the proxalutamide and remdesivir combination (Fig. 3 *E* and *F*). Both proxalutamide or enzalutamide and remdesivir combination treatments had no detrimental effects on the viability of iAEC2 cells (Fig. 3 *D* and *G*). These results suggest that proxalutamide may have clinical utility in combination with current SARS-CoV-2 treatments, such as remdesivir.

**Fig. 4.**
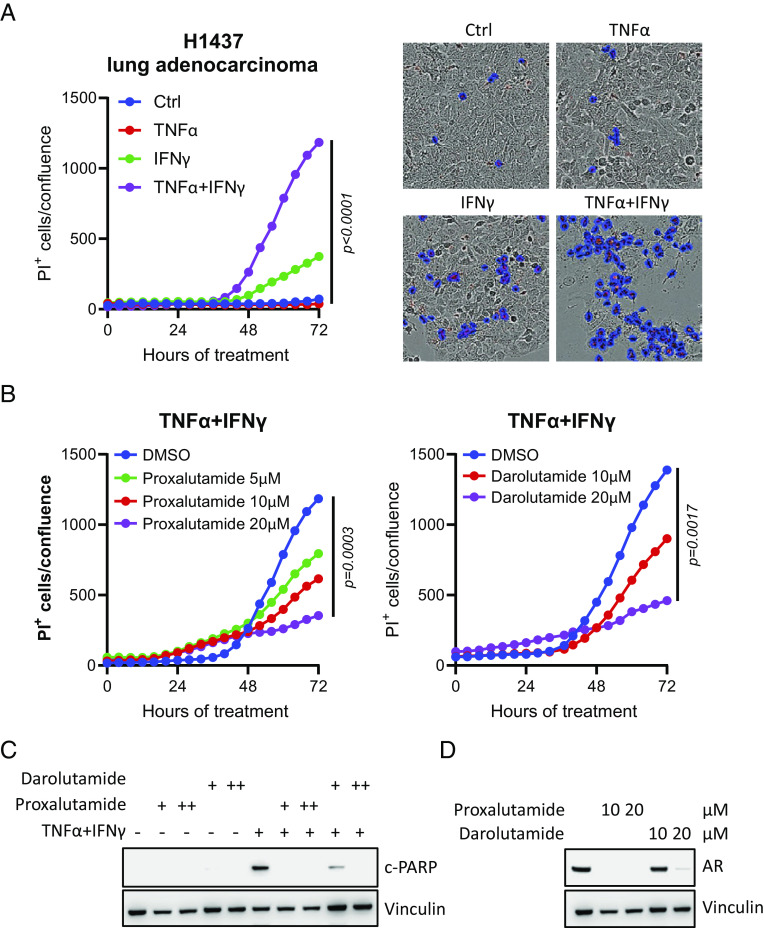
Proxalutamide attenuates CSS–related cell death and mortality. (*A*) Real-time analysis of cell death in H1437 cells in vitro under control, TNFα, IFNγ, or combination treatment. Representative images of dead cells under the indicated conditions are shown on the *Right*. The *P* value was calculated by the two-tailed unpaired *t* test between control and TNFα/IFNγ combination treatment. (*B*) Real-time analysis of cell death in H1437 cells in vitro under TNFα and IFNγ combination and various concentrations of proxalutamide or darolutamide. *P* values were calculated by the two-tailed unpaired *t* test comparing dimethylsulfoxide (DMSO) control and 20 µM proxalutamide or darolutamide. (*C*) Immunoblotting of c-PARP and vinculin (loading control) in H1437 cells after treatment with 10 and 20 µM of proxalutamide or darolutamide with or without TNFα and IFNγ combination for 72 h. (*D*) Immunoblotting of AR and vinculin in H1437 after treatment with 10 and 20 µM of proxalutamide or darolutamide for 72 h.

SARS-CoV-2-induced mortality is largely triggered by a cytokine storm that occurs in the pulmonary system and systemically (53). It has been reported that TNFα and INFγ can act synergistically to trigger inflammatory cell death in vitro and in vivo, which mimics the SARS-CoV-2-induced cytokine shock syndrome (CSS) that occurs in COVID-19 patients (50). Specifically, TNFα and INFγ induce a type of inflammatory cell death called PANoptosis, which is regulated by the PANoptosome and involves molecular components of pyroptosis, apoptosis, and necroptosis (50, 54). In an AR-positive lung cell line, H1437, we demonstrated that the combination of TNFα and INFγ induced maximal cell death compared to either cytokine alone (Fig. 4*A*). Interestingly, the cell death induced by combination treatment with TNFα and INFγ was attenuated by proxalutamide and another AR antagonist darolutamide in a dose-dependent manner (Fig. 4*B*) but not by enzalutamide or apalutamide (*SI Appendix*, Fig. S1*A*). Additionally, the cell death triggered by TNFα and INFγ combination treatment was confirmed by elevated cleaved PARP (c-PARP) levels, which were dose dependently blocked by proxalutamide and darolutamide (Fig. 4*C*) but not enzalutamide or apalutamide (*SI Appendix*, Fig. S1*B*). Similarly, AR protein levels were down-regulated by proxalutamide and darolutamide (Fig. 4*D*) but not enzalutamide or apalutamide (*SI Appendix*, Fig. S1*C*). This suggests that AR antagonists such as proxalutamide or darolutamide may provide additional benefits in terms of reducing CSS in vivo. In normal mouse prostate organoids, we confirmed that proxalutamide inhibited murine AR signaling by decreasing androgen (dihydrotestosterone, DHT)-stimulated induction of *Fkbp5* and *Psca* target genes; additionally, proxalutamide decreased *Ar* mRNA levels (*SI Appendix*, Fig. S2*A*). These results prompted us to examine the in vivo efficacy of proxalutamide in preventing death in the TNFα and INFγ CSS model (50) in wild-type C57BL6 male mice. We tested two treatment regimens of proxalutamide prior to cytokine challenge with the TNFα and INFγ combination. The data showed that both proxalutamide treatment regimens reduced mortality induced by TNFα and INFγ (*SI Appendix*, Fig. S2 *B* and *C*). Histology evaluation of tissue damage triggered by TNFα and INFγ combination was examined in the small intestine and lung (*SI Appendix*, Fig. S2*D*). Compared with the PBS treated group, atrophy of the villi and an increase in inflammatory cell infiltration in the lamina propria area of the intestine were observed post-TNFα and IFNγ treatment, which was largely alleviated with proxalutamide treatment. In addition, TNFα and IFNγ treatment induced interlobular septal thickening in the lungs of mice showing focal epithelial hyperplasia, and such effects were rescued by proxalutamide treatment. Thus, these results suggest that proxalutamide may reduce TNFα and IFNγ cytokine storm-induced cell death in vitro and in vivo.

**Fig. 5.**
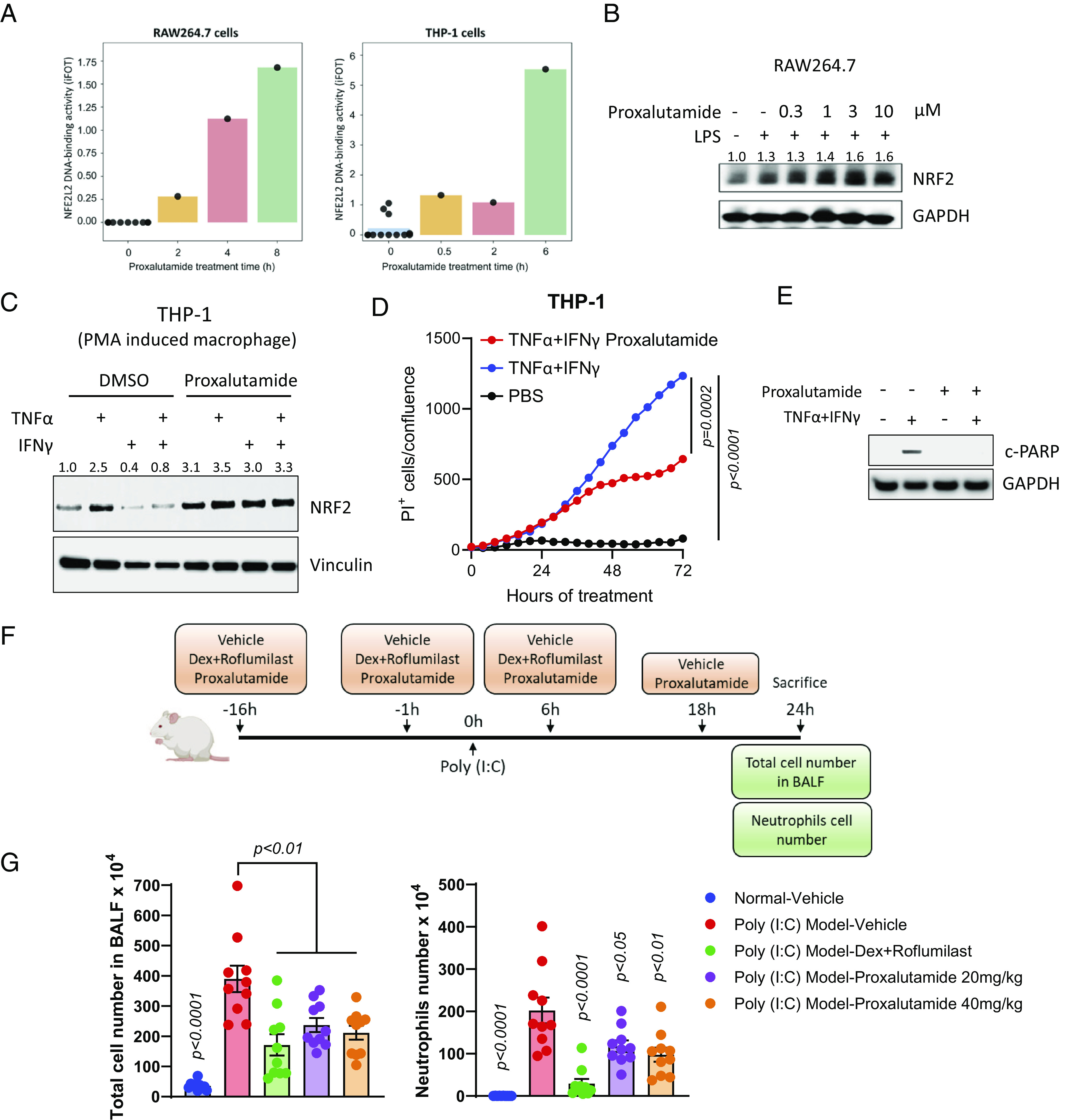
Proxalutamide enhances NRF2 transcriptional activity and inhibits acute immune response in the poly (I:C)-induced lung injury animal model. (*A*) Proxalutamide increased NRF2 transcriptional activity in RAW264.7 and THP-1 cells. (*B*) Immunoblotting of NRF2 protein in RAW264.7 cells with or without LPS stimulation and indicated concentration of proxalutamide. GAPDH serves as a loading control. (*C*) Immunoblotting of NRF2 protein in THP-1 cells with TNFα, IFNγ, or combination, with or without 20 µM proxalutamide. Vinculin serves as a loading control. (*D*) Real-time analysis of cell death in THP-1 cells in vitro treated with the indicated cytokines. *P* values were calculated by the two-tailed unpaired *t* test between the indicated groups. (*E*) Immunoblotting of c-PARP and GAPDH in THP-1 cells after treatment with proxalutamide with or without TNFα and IFNγ combination for 72 h. (*F*) Schematic illustration of acute immune response in poly (I:C)-induced acute lung injury animal model. (*G*) Total cell number and neutrophil cell counts in the bronchoalveolar lavage fluid (BALF) under indicated treatment. *P* values were calculated by the two-tailed unpaired *t* test between the poly (I:C)-vehicle and indicated treatment.

The NRF2 pathway is an important part of cellular defense through the production of antioxidants, which occurs via binding of the NRF2 transcription factor to antioxidant response elements in target genes (55–57). The upregulation of NRF2 has been reported to control inflammation in several studies (56–60). Here, we found that proxalutamide increases NRF2 transcriptional activity by enhancing NRF2 DNA binding in RAW264.7 and THP-1 cells (Fig. 5*A*). In RAW264.7 cells, proxalutamide also up-regulated NRF2 protein expression in lipopolysaccharide (LPS)-stimulated conditions (Fig. 5*B*). In the in vitro CSS model triggered by TNFα and INFγ combination treatment, proxalutamide augmented NRF2 protein levels and decreased cell death in THP-1 cells (Fig. 5 *C* and *D*). Apoptotic cell death triggered by TNFα and INFγ combination treatment was attenuated by proxalutamide (Fig. 5*E*). Next, we examined proxalutamide in an acute lung injury animal model triggered by poly(I:C), and combination dexamethasone and roflumilast treatment was used as a positive control (Fig. 5*F*). In this model, proxalutamide significantly reduced the total mononuclear cells and neutrophils in alveolar lavage fluids from poly(I:C)-induced animals (Fig. 5*G*). Together, our data show that proxalutamide up-regulates NRF2 protein levels and decreases inflammation in the lungs induced by poly(I:C), suggesting a possible benefit of proxalutamide against SARS-CoV-2-associated inflammatory responses and mortality in COVID-19 patients.

## Discussion

Proxalutamide was initially developed as an AR antagonist that could potentially have efficacy in CRPC patients, including those that had developed resistance to existing AR-targeted therapies. Results from phase 1 testing in CRPC patients showed that proxalutamide was well tolerated, had a favorable pharmacokinetic profile, and exhibited antitumor activity in select patients (47). AR-targeting compounds became one of the initial groups of drugs to be pursued as potential COVID-19 treatments for the myriad of reasons discussed in preceding sections. With phase 1 testing complete, proxalutamide was positioned to be tested in the setting of COVID-19, along with other AR-targeted drugs that have been FDA-approved for prostate cancer for years, such as enzalutamide. Although positive results were reported for the initial COVID-19 trials with proxalutamide, clarity is still needed as one of the studies was retracted last year (41–44). Here, we performed several in vitro and in vivo assays assessing the activity of proxalutamide against SARS-CoV-2 infection and inflammatory responses. We indeed demonstrate that proxalutamide decreases SARS-CoV-2 infectivity in vitro, and the compound is active against several strains of the virus (WA1, alpha, delta, and omicron). Synergy can be obtained when proxalutamide is combined with remdesivir. Interestingly, proxalutamide also increases levels of the NRF2 transcription factor.

It is well established that COVID-19 can be associated with a cytokine storm, a hyperactivation of the immune system that can ultimately result in death (53). In this study, we employed two in vivo lines of experimentation to analyze the effect of proxalutamide on CSS and lung injury. Proxalutamide pretreatment in the TNFα/IFNγ model of CSS (50) results in a modest increase in overall survival (*SI Appendix*, Fig. S2 *B* and *C*), mirroring the attenuation of in vitro cell death observed with proxalutamide in the H1437 and THP-1 cell lines (Figs. 4*B* and 5*D*). Using poly(I:C) that induces inflammatory responses in the lung similar to viral infections (61), we observe that proxalutamide significantly decreases total cell and neutrophil levels in BALF (bronchoalveolar lavage fluid) (Fig. 5*G*). Altogether, results from these two in vivo models suggest that proxalutamide can decrease CSS responses and lung inflammation, but there are associated caveats to note. TNFα and IFNγ induce PANoptosis in mice that leads to CSS and death, which has been suggested to mimic severe COVID-19 in patients (50). However, TNFα/IFNγ-induced death in mice occurs within hours, whereas death from acute respiratory distress syndrome (ARDS) in COVID-19 patients happens over a much longer time (62). Additionally, studies have implicated alternative cytokines (e.g., IL-6 and IL-1) rather than just TNFα and IFNγ as the primary inducers of ARDS in COVID-19 (63). In terms of the poly(I:C) model, it is prudent to also note that this is a model of lung injury, rather than lung epithelial cell death. Finally, these in vivo experiments are models of the possible downstream effects of SARS-CoV-2 and did not directly involve animal infection with the virus. It is interesting to note, however, that proxalutamide increases the DNA binding activity and expression of Nrf2, and Nrf2 has been shown to be an essential factor for tempering the immune response and protecting against sepsis (64, 65). A recent study also shows that SARS-CoV-2 can inhibit Nrf2 signaling through one of its nonstructural proteins (66). In line with our findings, Nrf2 agonists consequently inhibited SARS-CoV-2 replication (66).

Combined, the data in this study support the notion that proxalutamide has antiviral activity against SARS-CoV-2 and suggest that it could show positive clinical benefit in cases of COVID-19, warranting further clinical exploration. In comparison, as mentioned above, clinical studies with degarelix (HITCH trial, NCT04397718) and enzalutamide (COVIDENZA trial, NCT04475601) did not find any improvements in clinical outcome with COVID-19 (39, 40). There are a multitude of explanations that could account for these disparate findings from different AR-targeting drugs. Degarelix is a GnRH antagonist that prevents release of follicle-stimulating hormone and luteinizing hormone, thereby leading to suppression of testicular testosterone release and a decrease in AR activity at the level of ligand availability (67). In contrast, proxalutamide, like enzalutamide, binds directly to the ligand-binding domain of AR to block receptor activation (47, 68). As shown in Fig. 1, proxalutamide and enzalutamide exert similar effects in LNCaP prostate cancer cells—decreasing or activating similar signaling pathways, decreasing androgen signaling, and decreasing cell proliferation. Relevant to SARS-CoV-2, both compounds decrease expression of host entry receptors *ACE2* and *TMPRSS2* (Fig. 1*F*). However, certain differences exist with these two compounds. For instance, a preclinical report on proxalutamide reported a 3.4-fold higher binding affinity for AR compared to enzalutamide (47). As shown here and previously (47), proxalutamide can also decrease AR protein expression, while enzalutamide does not lead to AR degradation (Fig. 1*E*). In the SARS-CoV-2 bioassays, proxalutamide exhibited increased potency in inhibiting infection compared to enzalutamide (IC_50_ of 97 nM for proxalutamide and 281 nM for enzalutamide, Fig. 2*B*) and a higher Bliss synergy score with remdesivir (14.516 and 11.685 for proxalutamide and enzalutamide, respectively, Fig. 3). Furthermore, in the cell line models of cytokine-mediated death with combined TNFα and IFNγ treatment, addition of proxalutamide prevented cell death (Fig. 4*B*), whereas enzalutamide was without effect, even at the high dose of 20 µM (*SI Appendix*, Fig. S1*A*). These data show that although proxalutamide and enzalutamide are both AR antagonists, differences in their mechanisms of action exist. However, since both compounds decrease *ACE2* and *TMPRSS2* expression and ultimately prevent SARS-CoV-2 infectivity in vitro (albeit with different IC_50_ values), further research is needed to define the precise mechanisms that could account for disparate clinical outcomes in COVID-19 treatment.

Several phase 3 clinical trials of proxalutamide treatment for COVID-19, all sponsored by Kintor Pharmaceuticals, are ongoing in different countries, and these studies should provide more definitive answers as to its efficacy. One phase 3 randomized, placebo-controlled, multiregional clinical trial of outpatients with mild or moderate COVID-19 (NCT04870606) primarily enrolled patients at centers across the United States (99%) (69). Efficacy data showed that proxalutamide reduced the risk of hospitalization or death compared to placebo, and proxalutamide continued to show a positive safety profile (69). An additional outpatient clinical trial of males with mild to moderate COVID-19 in Brazil is ongoing (NCT04869228), with the primary outcome being oxygen requirement at Day 28. Finally, NCT05009732 is an ongoing phase 3 trial of proxalutamide in hospitalized adults with COVID-19 that has participating locations across several countries, including the United States, China, Philippines, and South Africa. The primary end point for this study is time to clinical deterioration (need for ICU care, mechanical ventilation, or mortality). The data presented in our report suggest that proxalutamide can markedly decrease SARS-CoV-2 infectivity and associated inflammatory responses, which could result in positive clinical benefit, and results from the clinical studies above are eagerly awaited.

## Methods

### Cell Culture.

LNCaP, RAW264.7, and THP-1 cells were purchased from the American Type Culture Collection (ATCC) and cultured in 5% CO_2_ at 37 °C in medium as suggested by ATCC. iAEC2 cells [iPSC (SPC2 iPSC line, clone SPC2-ST-B2, Boston University) derived alveolar epithelial type 2 cells] were maintained as previously described (52). iAEC2 cells were also subcultured as previously described (70). Cell lines underwent genotype authentication and were confirmed to be negative for mycoplasma.

### SARS-CoV-2 Bioassay.

SARS-CoV-2 isolates USA-WA1/2020, hCoV-19/USA/OR-OHSU-PHL00037/2021 (Lineage B.1.1.7; Alpha Variant), hCoV-19/USA/MD-HP05285/2021 (Lineage B.1.617.2; Delta Variant), and hCoV-19/USA/GA-EHC-2811C/2021 (Lineage B.1.1.529; Omicron Variant) were obtained from BEI resources and propagated in VeroE6 cells (ATCC). Viral titers were established by TCID50 with the Reed and Muench method. LNCaP or iACE2 cells were plated in 384-well plates and treated with increasing concentrations of proxalutamide or enzalutamide for 24 h prior to SARS-CoV-2 virus infection in a Biosafety Level 3 facility. Cells were then incubated for 48 h postinfection under culture conditions of 5% CO_2_ and 37°C. Assay plates were fixed, permeabilized, and labeled with antinucleocapsid SARS-CoV-2 primary antibody (Antibodies Online, Cat. #: ABIN6952432) as previously described (52). The remaining of the assay proceeded as previously described (70).

### Fluorescence Imaging and High-Content Analysis.

A Thermo-Fisher CX5 high-content microscope with LED excitation (386/23 nm, 650/13 nm) at 10× magnification was used to image assay plates. Nine fields per well were imaged at a single Z-plane in these experiments. Imaging, processing, and normalization were performed as previously described (70, 71).

### Gene Expression Analysis.

RNA was extracted from LNCaP cells treated with DMSO, 20 µM proxalutamide, or enzalutamide for 8 h using a Qiagen RNA extraction kit. RNA quality was determined using a Bioanalyzer RNA Nano Chip. Poly-A selection was performed with Sera-Mag Oligo(dT)-Coated Magnetic Particles (38152103010150; GE Healthcare Life Sciences), and libraries were generated using a KAPA RNA HyperPrep kit (KK8541; Roche Sequencing Solutions). RNA-seq was performed on an Illumina HiSeq 2500. Reads were aligned with the Spliced Transcripts Alignment to a Reference mapper to the human reference genome gh38. Gene differential expression analysis was carried out with edgeR70.

### Mouse Prostate Organoid Culture.

Whole mouse prostate was dissected from C57BL6J wild-type mice, and organoid culture was generated according to previous publication (72). Mouse prostate organoids were treated with 5 µM or 10 µM proxalutamide or enzalutamide for 16 h prior to 10 nM DHT stimulation for 8 h. Total RNA was extracted from organoid culture using the miRNeasy mini kit (Qiagen), and cDNA was synthesized from 1 µg total RNA using the High-Capacity cDNA Reverse Transcription Kit (Applied Biosystems). qPCR was performed using fast SYBR green master mix on the QuantStudio Real-Time PCR Systems (Applied Biosystems). The SYBR green primer sequences are *Fkbp5* forward: GATTGCCGAGATGTGGTGTTCG, *Fkbp5* reverse: GGCTTCTCCAAAACCATAGCGTG; *Psca* forward: GCACAGTTGCTTTACATCGCGC, *Psca* reverse: ACAGGTCAGAGTAGCAGCACGT; and *Ar* forward: CCTTGGATGGAGAACTACTCCG, *Ar* reverse: TCCGTAGTGACAGCCAGAAGCT.

### Immunoblotting.

For western blotting analysis, cells were harvested and lysed in Pierce RIPA buffer (Thermo Fisher) with added phosphatase (Millipore) and protease (Roche) inhibitor cocktails. Protein quantification, sodium dodecyl-sulfate polyacrylamide gel electrophoresis, transfer, blocking, and antibody incubation were performed as described previously (73), and protein signals were detected with ECL Primer (Amersham) on a Li-Cor machine. Antibodies were used at dilutions recommended by the manufacturer and consisted of the following: AR (06-680, Millipore), PSA (Dako), NRF2 (12721S, Cell Signaling Technology), and GAPDH (3683S, Cell Signaling Technology).

### Real-Time Imaging for Cell Death.

The kinetics of cell death were determined using the IncuCyte ZOOM (Essen BioScience) live-cell automated system. H1437 or THP-1 cells (1 × 10^5^ cells/well) were seeded in 24-well tissue culture plates. Cells were treated with 50 ng/mL of human TNFα (Peprotech, AF-300-01A) and /or 100 ng/mL of human IFNγ (Peprotech, 300-02) for the indicated time and stained with 1 µg/mL propidium iodide (PI) (Life Technologies, P3566) following the manufacturer’s protocol. The plate was scanned, and fluorescent and phase-contrast images were acquired in real-time every 4 h. PI-positive dead cells are marked with a red mask for visualization. The image analysis, masking, and quantification of dead cells were done using the software package supplied with the IncuCyte imager.

### In Vivo TNFα and IFNγ-Induced Inflammatory Shock.

C57BL6J mice were purchased from The Jackson Laboratory. Eight- to nine-week-old male C57BL6J mice were given vehicle or 40 mg/kg proxalutamide by oral gavage either 2 h or once daily for 5 d prior to cytokine injection. Cytokine combination of 10 μg TNFα (Preprotech, 315-01A) and 20 μg IFNγ (Preprotech, 315-05) was diluted in Dulbecco’s phosphate-buffered saline (PBS) and injected intraperitoneally. After cytokine injection, animals were under permanent observation, and survival was assessed every 30 min.

### Poly(I:C)-Induced Acute Lung Injury In Vivo Model.

Six- to eight-week-old male BALB/c (Bagg Albino/c) mice were assigned to treatment groups by randomization in BioBook software to achieve similar group mean weight before treatment; 10 mice were allocated into each group. Group 1 was normal-vehicle; groups 2 to 5 were challenged with poly(I:C) with vehicle sodium carboxymethly cellulose (CMC-Na), 10 mg/kg dexamethasone and 20 mg/kg roflumilast combination, 20 mg/kg proxalutamide, or 40 mg/kg proxalutamide, respectively. Dexamethasone was dissolved in 0.5% CMC-Na to make a suspension at a final concentration of 1 mg/mL. Roflumilast was dissolved in 0.5% CMC-Na to make a suspension at a final concentration of 2 mg/mL. Mice were treated with vehicle, dexamethasone and roflumilast combination, or proxalutamide 16 h and 1 h prior to poly(I:C) injection and 6 h after poly(I:C) injection. Additional proxalutamide dose was given 18 h post poly(I:C) injection. Poly(I:C) solution was prepared to a 0.06% solution in sterile PBS freshly prepared where 1.8 mg poly(I:C) was dissolved in 3 mL PBS to make a suspension at a final concentration of 0.6 mg/mL. Twenty-four hours post poly(I:C) injection, all mice were anesthetized with Zoletil (i.p., 25 to 50 mg/kg, containing 1 mg/mL Xylazine). Lungs were gently lavaged via the tracheal cannula with 0.5 mL PBS containing 1% fetal bovine serum (FBS), and the BALF was collected. Then, the lungs were gently lavaged with another 0.5 mL PBS containing 1% FBS. After lavage, the collected BALF was stored on ice. The total cell number in BALF was counted using a hemocytometer. After lavage by PBS, all mice were killed by exsanguination.

### Liquid Mass Spectrometry Quantification after TFRE (Transcription Factors Response Element) Enrichment.

Mouse monocyte RAW264.7 cells (0, 2 h, 4 h, and 8 h) and human monocyte THP-1 (0, 0.5 h, 2 h, and 6 h) were treated with 10 μM proxalutamide, respectively. Cells were collected and cocultured with TFRE-binding beads, and the beads were rotated and combined for 1.5 h at 4°C. After the combined TFRE beads were washed 3 times with NETN and 2 times with mass spectrometry (to remove the scale removing agent; if there were still bubbles, they were washed again with water). Then, 50 μL NH_4_HCO_3_ and 1.5 μg tyrosinase were added to the beads. The beads were hydrolyzed overnight, and the tube wall was lightly spritzed 1 to 2 times in the middle. Two hundred microliters of 50% acetonitrile + 0.1% formic acid was added to the suspension for 3 to 5 min, and then, the supernatant was transferred on a magnetic rack to a new Eppendorf tube; this was then repeated once. The supernatant was vacuum dried into peptide powder and stored at low temperature. Protein sequences were identified by liquid chromatography with tandem mass spectrometry.

### Statistical Analysis.

Statistical analyses were performed by the two-tailed, unpaired *t* test, unless otherwise indicated in figure captions. Error bars indicate mean ± SEM. GraphPad Prism software (version 9) was used for statistical calculations. No data were excluded from the analyses.

## Supplementary Material

Appendix 01 (PDF)

## Data Availability

All study data are included in the article and/or *SI Appendix*. Sequencing data are available through the National Center for Biotechnology Information Gene Expression Omnibus, accession number GSE234805 (74).
