# Supplementary material for: Proxalutamide reduces SARS-CoV-2 infection and associated inflammatory response
Source: Proc Natl Acad Sci U S A. 2023 Jul 17;120(30):e2221809120. doi: 10.1073/pnas.2221809120 (PMC10372636; doi:10.1073/pnas.2221809120)
Supplement: Supplementary file 1 — Appendix 01 (PDF) [file pnas.2221809120.sapp.pdf]

PNAS

Supporting Information for
Proxalutamide reduces SARS-CoV-2 infection and associated inflammatory response

Yuanyuan Qiao[1,2,3*], Jesse W. Wotring[4*], Yang Zheng[1*], Charles J. Zhang[4], Yuping Zhang[1,2], Xia Jiang[1], Carla D. Pretto[5], Sanjana Eyunni[1], Abhijit Parolia[1], Tongchen He[1], Caleb Cheng[1], Xuhong Cao[1], Rui Wang[1], Fengyun Su[1], Stephanie J. Ellison[1], Yini Wang[6], Jun Qin[6], Honghua Yan[7], Qianxiang Zhou[7], Liandong Ma[7], Jonathan Z. Sexton[4,5,8,9,10,#], and Arul M. Chinnaiyan[1,2,3,11,12,#]

[1] Michigan Center for Translational Pathology, University of Michigan, Ann Arbor, MI, 48109, USA
[2] Department of Pathology, University of Michigan, Ann Arbor, MI, 48109, USA
[3] Rogel Cancer Center, University of Michigan, Ann Arbor, MI, 48109, USA
[4] Department of Medicinal Chemistry, College of Pharmacy, University of Michigan, Ann Arbor, MI, 48109, USA
[5] Department of Internal Medicine, University of Michigan, Ann Arbor, MI, 48109, USA
[6] State Key Laboratory of Proteomics, Beijing Proteome Research Center, National Center for Protein Sciences (Beijing), Beijing Institute of Lifeomics, Beijing, 102206, China
[7] Kintor Pharmaceutical Limited, No. 20 Songbei Road, Suzhou Industrial Park, Jiangsu, 215123, China
[8] Center for Drug Repurposing, University of Michigan, Ann Arbor, MI, 48109, USA
[9] Michigan Institute for Clinical and Health Research, University of Michigan, Ann Arbor, MI, 48109, USA
[10] Department of Pharmacology, University of Michigan, Ann Arbor, MI, 48109, USA
[11] Howard Hughes Medical Institute, University of Michigan, Ann Arbor, MI, 48109, USA
[12] Department of Urology, University of Michigan, Ann Arbor, MI, 48109, USA
* These authors contributed equally
[#] Co-senior authors

Correspondence to:
Arul M. Chinnaiyan, M.D., Ph.D.
arul@med.umich.edu

This PDF file includes:

Figure S1
Figure S2

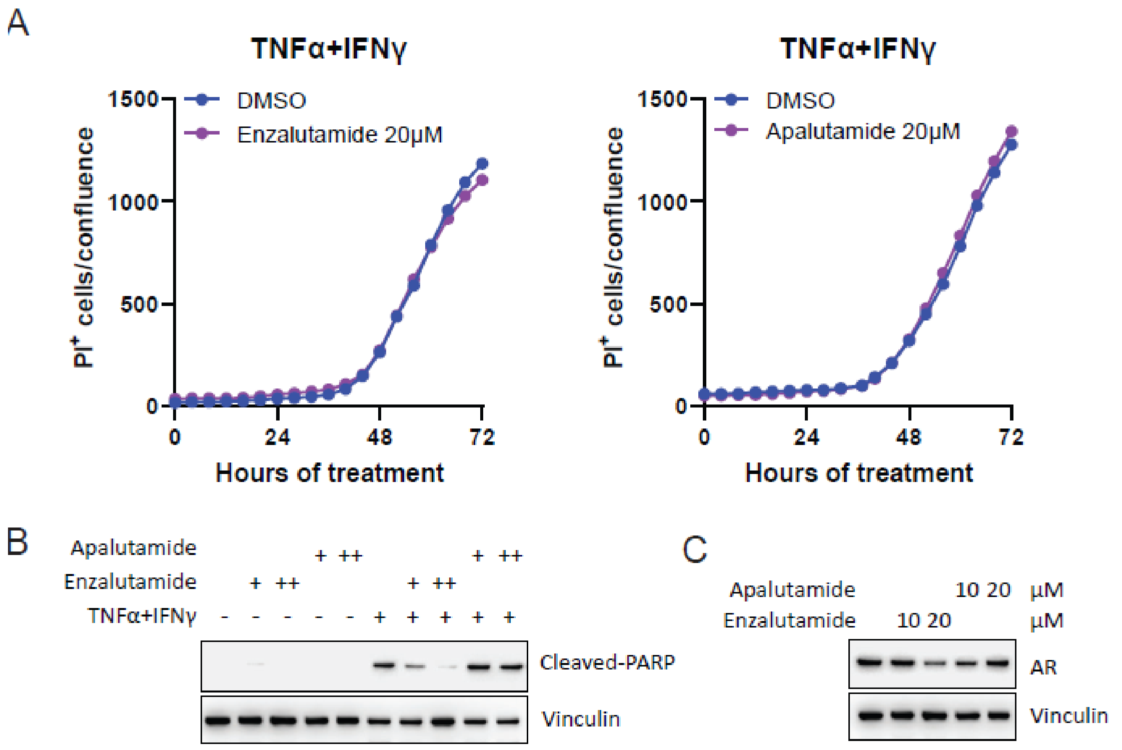

Fig. S1. Enzalutamide and apalutamide have no effect on reducing cytokine shock syndrome-related cell death and AR protein in H1437 cells.

A, Real-time analysis of cell death in H1437 cells *in vitro* under TNFα and IFNγ combination and various concentrations of enzalutamide and apalutamide.

B, Immunoblotting of cleaved PARP and vinculin in H1437 cells after 72 hours treatment of 10 and 20 μM of enzalutamide or apalutamide with or without TNFα and IFNγ combination.

C, Immunoblotting of AR and vinculin in H1437 cells after 72 hours treatment of various concentrations of enzalutamide or apalutamide.

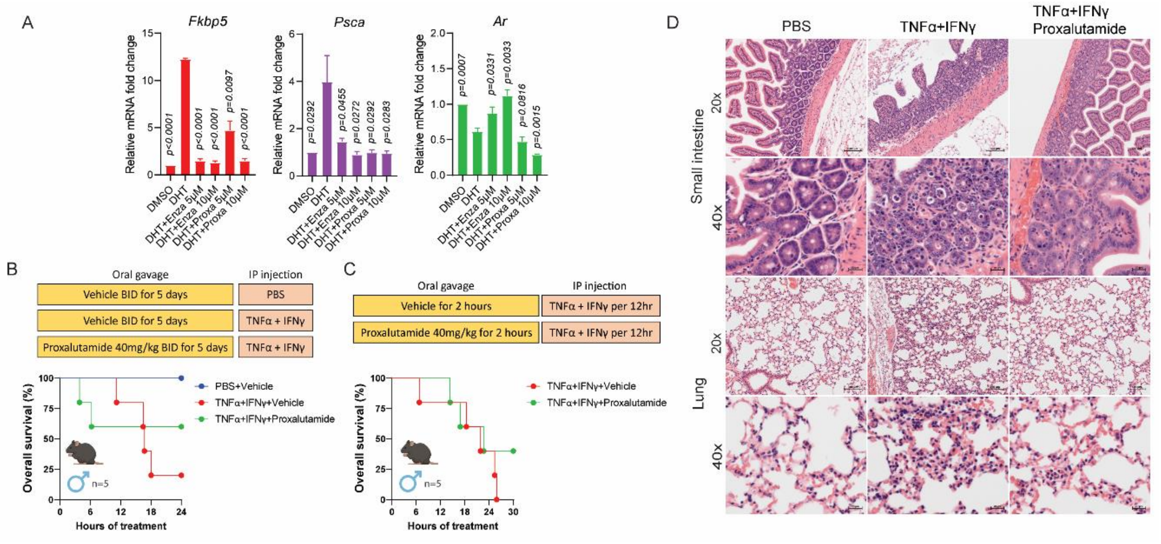

Fig. S2. Proxalutamide attenuates cytokine shock syndrome-related cell death and mortality.

A, Relative mRNA expression of *Fkbp5*, *Psca*, and *Ar* in mouse organoids upon the indicated treatment. P values were calculated by two-tailed unpaired t test for *Fkbp5*, and one-tailed unpaired t test for *Psca* and *Ar,* between the indicated treatment group and DHT.

B, Overall survival of *in vivo* cytokine storm model triggered by TNFα and IFNγ combination in C57BL6 male mice pretreated with either vehicle or 40 mg/kg proxalutamide for five days.

C, Overall survival of *in vivo* cytokine storm model triggered by TNFα and IFNγ combination in C57BL6 male mice with either vehicle or 40 mg/kg proxalutamide for two hours.

D, Representative H&E images of small intestine and lung after 5 hours treatment of PBS, TNFα and IFNγ combination, or TNFα and IFNγ and proxalutamide in C57BL6 male mice.
